# Symmetry-breaking charge separation: from charge generation to functional charge utilization

**DOI:** 10.1039/d6sc03733j

**Published:** 2026-07-07

**Authors:** Hui-Jun Zhang, Lijin Wang, Jiahao Wang, Jianbin Lin, Dongho Kim

**Affiliations:** a College of Chemistry and Chemical Engineering, MOE Key Laboratory of Spectrochemical Analysis and Instrumentation, Fujian Key Laboratory of Chemical Biology, Xiamen University Xiamen 361005 P. R. China jb.lin@xmu.edu.cn; b Department of Chemistry, Yonsei University Seoul 03722 Korea dongho@yonsei.ac.kr

## Abstract

Symmetry-breaking charge separation (SB-CS) has emerged as a promising route to ultrafast charge generation with minimal energy loss in strongly coupled chromophore dimers. However, despite sub-100 fs charge separation and near-unity efficiencies, its translation into long-lived and directional charge utilization remains a challenge. The difficulty arises from a fundamental mismatch: the same strong coupling and energetic degeneracy that enable efficient SB-CS also promote recombination and isotropic charge migration. Here, we propose that SB-CS should be viewed not as an isolated photophysical event, but as a hierarchical process spanning multiple length scales. From this perspective, we establish a unified design framework built on three interdependent dimensions: symmetry origin, which governs deterministic charge localization; kinetic asymmetry, which decouples charge separation from recombination; and spatial organization, which enables directional charge transport. Within this framework, the functional unit extends beyond the dimer to spatially organized architectures in which charge generation, stabilization, and transport are distributed. This multiscale view transforms SB-CS from a photophysical phenomenon into a design paradigm for constructing functional charge flow, offering guiding principles for next-generation π-systems in photovoltaics, photocatalysis, and emerging quantum and spin-based applications.

## Abstract 2

Efficient charge separation alone does not guarantee functionality; instead, symmetry origin, kinetic asymmetry, and spatial organization collectively govern functional charge flow toward photovoltaics and photocatalysis.

## Graphical abstract

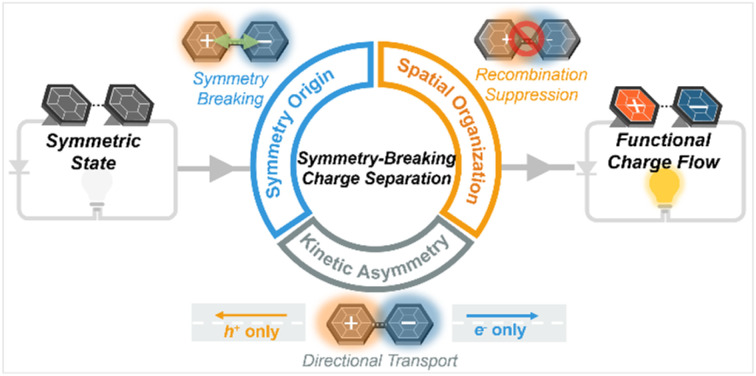

## 1 The SB-CS paradox: why efficient charge separation fails to deliver functionality

**Fig. 1:**
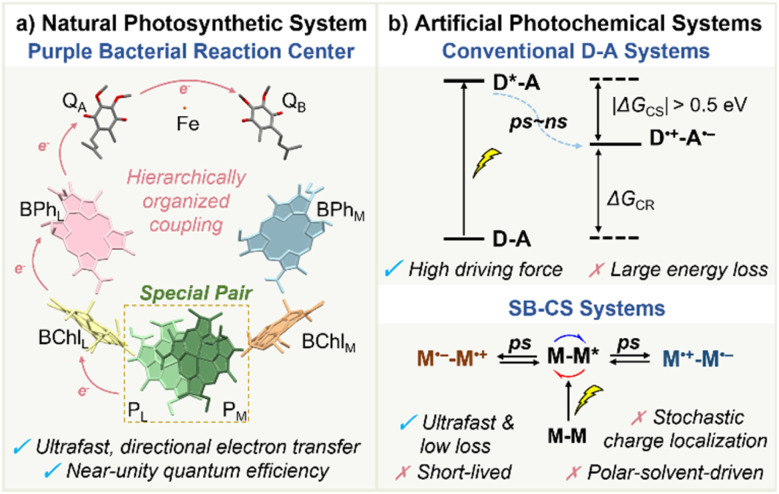
(a) Natural *versus* (b) artificial charge separation: from functional blueprint to photophysical limitations. In conventional D–A systems, the large free-energy change for charge separation (|Δ*G*_CS_| > 0.5 eV) results in substantial energy loss, limiting the energetic quality of the generated charges. SB-CS is a light-driven process in which electronically identical chromophores generate a charge-separated state through excited-state symmetry breaking, while retaining much of the absorbed photon energy.

Efficient photoinduced charge separation lies at the heart of solar energy conversion, photocatalysis, and molecular electronics. Natural photosynthetic reaction centers achieve this with near-unity quantum efficiency by orchestrating ultrafast, multistep electron transfer processes that not only generate charges, but also stabilize and direct them toward productive chemical outcomes (Fig. 1a).^1^ In contrast, artificial systems often face a fundamental trade-off: achieving rapid charge separation typically requires substantial thermodynamic driving forces, which can compromise the energetic quality of the resulting charges (Fig. 1b, for conventional donor–acceptor (D–A) systems).^2^

Symmetry-breaking charge separation (SB-CS) offers a compelling alternative (Fig. 1b, for SB-CS systems).^3^ By exploiting strong electronic coupling within symmetric or near-symmetric chromophore dimers (M–M in Fig. 1b), SB-CS can enable ultrafast charge separation^3*h*^ with relatively small energy losses (often on the order of 0.1–0.2 eV in optimized systems).^3*b*,*i*^ These features make SB-CS an attractive platform for next-generation optoelectronic and photonic materials.

However, despite extensive advances in understanding its photophysics, many SB-CS systems remain at the proof-of-concept stage with respect to functional charge utilization.

This apparent disconnect highlights an important consideration: efficient charge separation, while necessary, is not in itself sufficient for functional performance. In practice, even systems exhibiting sub-100 fs charge separation and near-unity quantum yields often fail to deliver long-lived or extractable charges.^4^

In many SB-CS systems, the same features that facilitate low-energy-loss charge separation, such as strong electronic coupling and energetic near-degeneracy, can also introduce challenges for charge localization, long-lived charge separation, and directional transport. Notably, these conditions typically correspond to coupling strengths in the range of tens to hundreds of meV, where excitonic delocalization enhances charge separation but also increases wavefunction overlap, thereby accelerating charge recombination (CR). Charge separation is often influenced by environmental fluctuations; charge-separated states are relatively short-lived; and charge migration may remain isotropic in the absence of additional bias.^5^

Addressing these challenges requires a shift in perspective. Rather than treating symmetry breaking as a primarily stochastic, environment-driven process, it may be more productive to consider how symmetry, kinetics, and spatial organization can be jointly encoded within supramolecular π-systems.^3*h*,6^ In this view, charge separation, stabilization, and transport are not independent objectives, but interconnected aspects of a unified design problem.

**Fig. 2:**
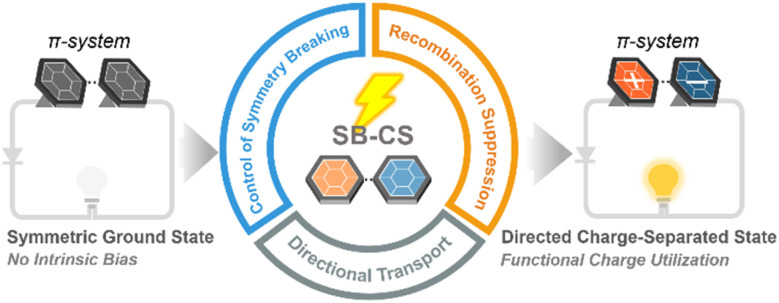
Integrated design framework for SB-CS: symmetry origin, kinetic decoupling, and directional emergence.

In this Perspective, we propose that advancing SB-CS toward functional systems benefits from adopting a reaction-center-inspired architectural framework. We organize recent developments along three interrelated design dimensions (Fig. 2): (i) the origin and control of symmetry breaking, (ii) the kinetic competition between charge separation and recombination, and (iii) the emergence of directionality from initially symmetric systems.

Building on these insights, we outline a conceptual blueprint for SB-CS architectures that aims to decouple charge generation from charge utilization through hierarchical organization and controlled coupling. This framework seeks to rationalize existing strategies while providing guiding principles for designing systems capable of efficient, long-lived, and directional charge separation across different environments. Ultimately, the goal is to move beyond demonstrating that SB-CS can generate charges, toward understanding how such charges can be stabilized, directed, and harnessed for function.

## 2 From stochastic symmetry breaking to intrinsic asymmetry

The initiation of SB-CS has traditionally been understood as an environmentally assisted process, in which solvent fluctuations transiently stabilize charge-localized states within otherwise symmetric chromophore assemblies.^7^ While this framework has been instrumental in establishing the fundamental photophysics of SB-CS, it also reveals an inherent limitation: symmetry breaking arises from stochastic environmental fluctuations, rendering charge localization intrinsically unpredictable and devoid of programmed directionality.^8^

This limitation exposes a more fundamental paradox: the same mechanism that enables charge separation inherently limits its controllability. In solvent-driven SB-CS, symmetry breaking is externally driven, yielding spatially indeterminate and non-directional charge flow.^3*d*,7*a*,9^ This lack of encoded bias is especially problematic in weakly polar or solid-state environments where solvent reorganization is suppressed.

Indeed, symmetry breaking must be designed as a structurally encoded function, where molecular architecture defines the pathway, directionality, and localization of charge separation. Accordingly, the evolution of SB-CS can be understood as a progression across three mechanistic regimes, reflecting an increasing degree of internal control over processes that were once governed by external fluctuations.

### 2.1 External symmetry breaking: environmental polarization

**Fig. 3:**
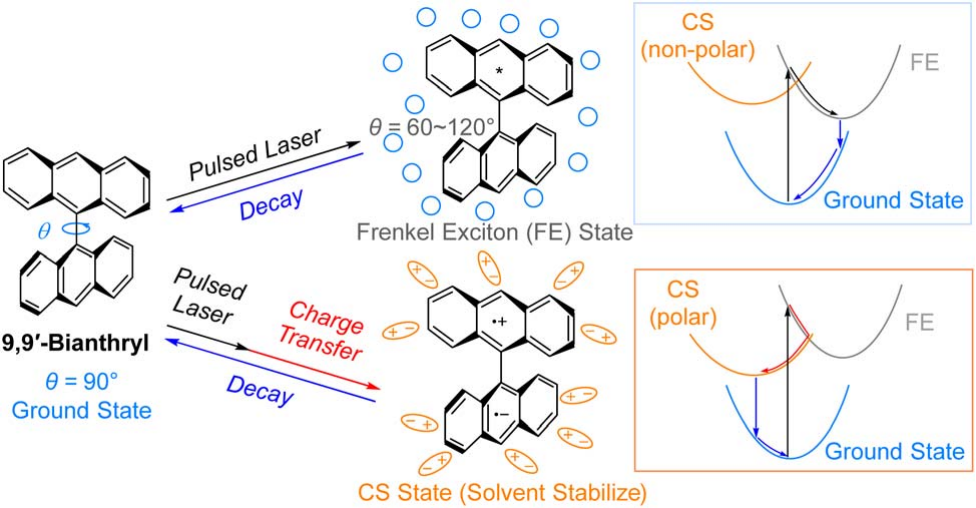
9,9′-Bianthryl (BA): a prototypical molecule for solvent-driven SB-CS. In nonpolar environments, the charge-separated state lies too high in energy to be accessed efficiently, leading primarily to exciton decay. In polar environments, dielectric stabilization lowers the energy of the CS state, enabling efficient SB-CS. Similar stabilization can also arise from static polarized environments in solid-state assemblies, highlighting the general role of environmental polarization in governing SB-CS.

The classical SB-CS paradigm is exemplified by symmetric dimers such as 9,9′-bianthryl (BA), where charge separation occurs only in sufficiently polar environments (Fig. 3).^3*c*,*f*,10^ In these systems, symmetry breaking is governed by a dynamic interplay of dielectric stabilization, solvent reorganization, and electronic configuration mixing. Time-resolved studies reveal that charge separation kinetics are strongly correlated with solvent relaxation times, indicating that the process is effectively “slaved” to environmental dynamics.^11^

Beyond solution-phase systems, recent studies have extended SB-CS to solid-state assemblies, where static environmental polarization can stabilize charge-separated states. In particular, Young and Wasielewski *et al.* demonstrated that the photophysics of BA single crystals can be tuned between singlet fission and SB-CS by controlling the polarity of solvent molecules intercalated within the crystal lattice during crystal growth.^12^ In crystals grown from highly polar benzonitrile, each BA molecule experiences an asymmetric local electrostatic environment because one anthracene unit is positioned directly adjacent to a benzonitrile molecule. This persistent environmental asymmetry selectively stabilizes charge-transfer configurations and enables ultrafast SB-CS within the instrument response time (∼300 fs), despite the absence of solvent motion. By contrast, solvent-free crystals lacking such electrostatic bias undergo rapid singlet fission instead. These observations highlight that the same physical mechanism, environmental polarization stabilizing charge-separated states, applies in both solution and solid-state systems, with the environment providing either dynamic or static polarization.

### 2.2 Structurally assisted symmetry breaking: dynamic amplification

A key advance beyond purely solvent-driven behavior is the recognition that nuclear motion is not merely a secondary response, but can act as the primary trigger of symmetry breaking.

**Fig. 4:**
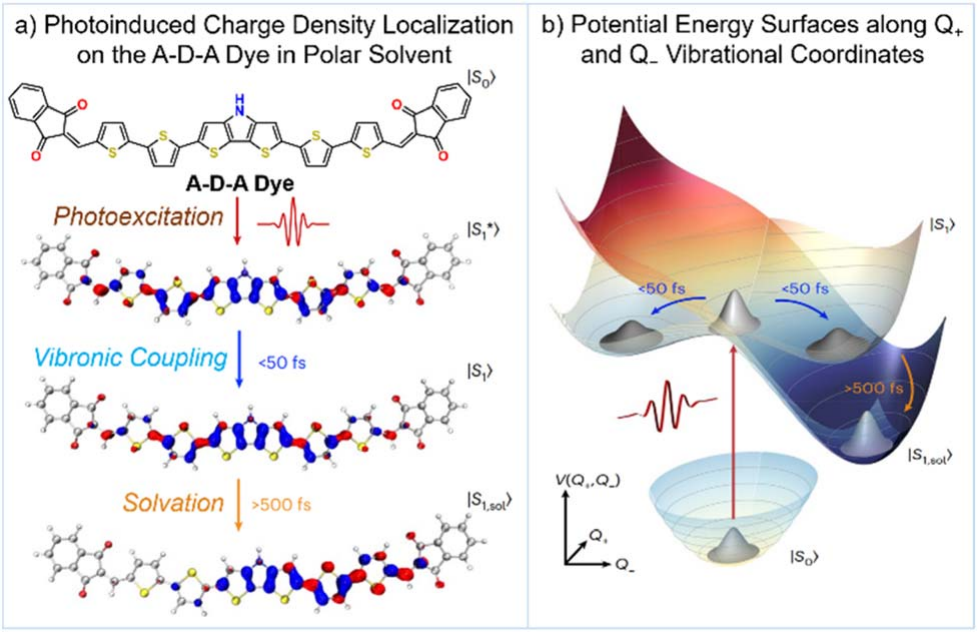
Vibronic coupling-driven symmetry breaking and subsequent solvation in a quadrupolar A–D–A dye. (a) Photoexcitation creates a symmetric, delocalized excited state 
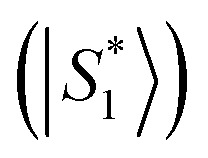
. (b) Vibronic coupling along the antisymmetric coordinate (*Q*_−_) generates a double-minimum potential, and polar solvent tilts this potential, leading to charge localization on one arm.

Ultrafast spectroscopic studies provide direct evidence for this mechanism. Using sub-10 fs two-dimensional electronic spectroscopy, De Sio, Lienau *et al.* demonstrated that vibronic coupling of an A–D–A dye initiates symmetry breaking within the first ∼50 fs (Fig. 4), significantly faster than typical solvent relaxation (≥1 ps), following photoexcitation, preceding any significant solvent response.^13^ High-frequency intramolecular vibrations (*e.g.*, 1400–1600 cm^−1^ C=C stretching modes) strongly couple to electronic states, generating a double-well potential along an antisymmetric vibrational coordinate. Notably, the early-time dynamics are nearly identical in nonpolar cyclohexane and polar dichloromethane, indicating that symmetry breaking at this stage is governed by intrinsic molecular motion rather than dielectric stabilization.

Following this initial triggering, the system evolves through a vibronically coherent regime. Within ∼200 fs, coherent coupling between Frenkel exciton (FE) and charge-transfer states promotes rapid state mixing, effectively amplifying the initial electronic asymmetry. As demonstrated by Hong *et al.*, this coherent superposition subsequently evolves toward charge localization as adiabaticity breaks down. On longer timescales (hundreds of fs to ps), structural fluctuations and solvent reorganization further stabilize the charge-separated state, with low-frequency intermolecular vibrations (∼200–400 cm^−1^) playing a dominant role in nonpolar environments.

This sequence defines a dynamically amplified symmetry-breaking mechanism, in which symmetry is first lifted by ultrafast vibrational motion, subsequently reinforced by vibronic coherence, and finally stabilized by slower nuclear and environmental degrees of freedom.^14^ Importantly, while this regime reduces reliance on solvent polarity, the outcome remains partially stochastic, as the ultimate localization pathway is not fully encoded in the molecular structure.

### 2.3 Intrinsic symmetry breaking: deterministic encoding

The ultimate limit of SB-CS design is reached when symmetry breaking is no longer dynamically generated, but instead pre-encoded in the molecular architecture. In this regime, structural asymmetry, arising from geometric arrangement, electronic differentiation, or local electrostatic fields, lifts degeneracy prior to photoexcitation, thereby predetermining the pathway of charge separation.

**Fig. 5:**
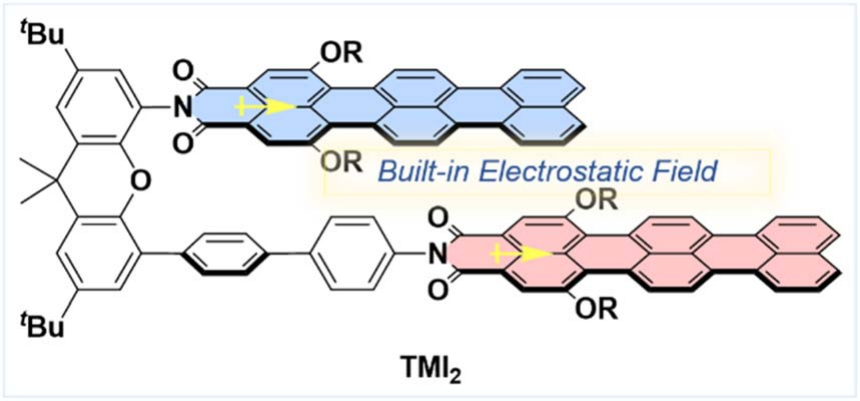
Intrinsic symmetry breaking *via* electrostatic encoding in a slip-stacked chromophore dimer TMI_2_: a built-in field enables solvent-independent SB-CS.

A compelling example of this concept is provided by the slip-stacked terrylene monoimide dimer TMI_2_ reported by Young and Wasielewski *et al.* (Fig. 5).^8*a*^ In contrast to conventional quadrupolar chromophores, each TMI unit carries a permanent dipole moment and supports an intramolecular charge-transfer state. When assembled in a head-to-tail slip-stacked configuration, these dipoles generate a built-in electrostatic field that pre-organizes asymmetry in the ground state. As a result, efficient SB-CS occurs even in nonpolar toluene with a near-zero driving force (Δ*G*_CS_ ≈ 0 eV), demonstrating that intrinsic electrostatic encoding can effectively replace solvent-induced stabilization.

This system exemplifies a key conceptual shift: symmetry breaking is no longer initiated by fluctuations, but is embedded in the molecular structure itself. In this sense, the system enters the excited state with symmetry already lifted, converting charge separation from a probabilistic event into a structurally biased process. However, TMI_2_ also highlights the limitations of partial asymmetry. Although the intrinsic dipoles of the TMI monomers generate an internal electrostatic field that biases SB-CS, the two charge-separated configurations remain nearly degenerate and no experimentally resolved site-specific charge localization has been demonstrated. Thus, the system exhibits biased rather than fully deterministic charge separation.

#### 2.3.1 Design principle: from fluctuations to architectures

**Fig. 6:**
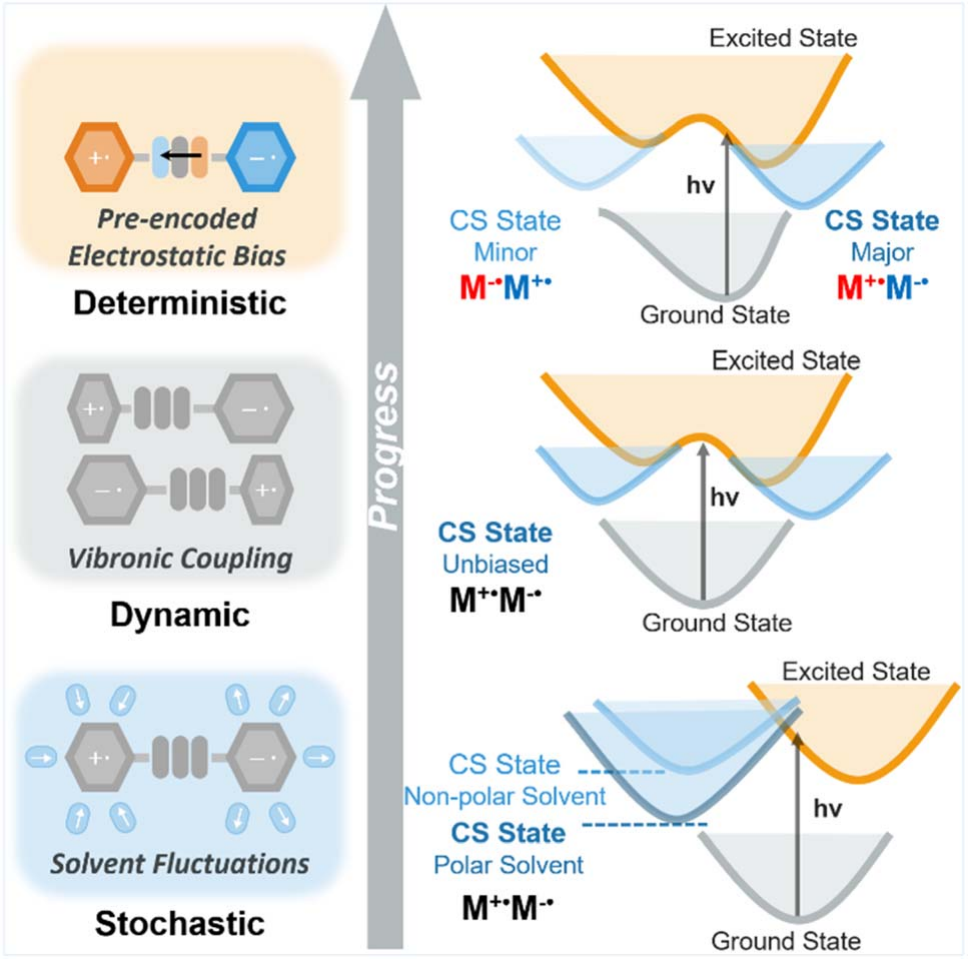
From fluctuations to architectures: progressive encoding of symmetry breaking in SB-CS.

Together, these three regimes reveal a unifying principle: functional SB-CS requires shifting the origin of symmetry breaking from external fluctuations to internal molecular architectures (Fig. 6), achieved through chemical asymmetry, geometric pre-organization, and electrostatic encoding, laying the foundation for the lifetime engineering and directional transport challenges discussed in the following sections.

## 3 Lifetime engineering through kinetic decoupling

While intrinsic symmetry breaking dictates where charges are initially generated, functional SB-CS systems must additionally ensure that these charges persist long enough to enable extraction, transport, or subsequent chemical transformations. However, the strong electronic interactions that enable ultrafast charge separation often simultaneously accelerate charge recombination, creating a kinetic trade-off. As a result, most SB-CS systems operate in a regime where forward and backward electron transfer processes remain intrinsically coupled, with charge-separated state lifetimes typically confined to the sub-nanosecond to nanosecond timescale. Such lifetimes are generally insufficient to support long-range charge transport or efficient interfacial charge extraction.

Extending these lifetimes therefore requires decoupling the forward and backward pathways. This can be achieved by independently controlling the physical parameters that govern each direction. Two distinct strategies have emerged: local kinetic control, which differentiates charge separation and recombination within a single molecular framework, and nonlocal kinetic control, which spatially separates charges through directed migration. In the following discussion, we distinguish between charge transfer (CT), referring to partially charge-separated or mixed Frenkel-exciton/CT states, and charge separation (CS), denoting the fully charge-separated state with electrons and holes localized on different chromophores.

### 3.1 Local kinetic control: three orthogonal design axes

**Fig. 7:**
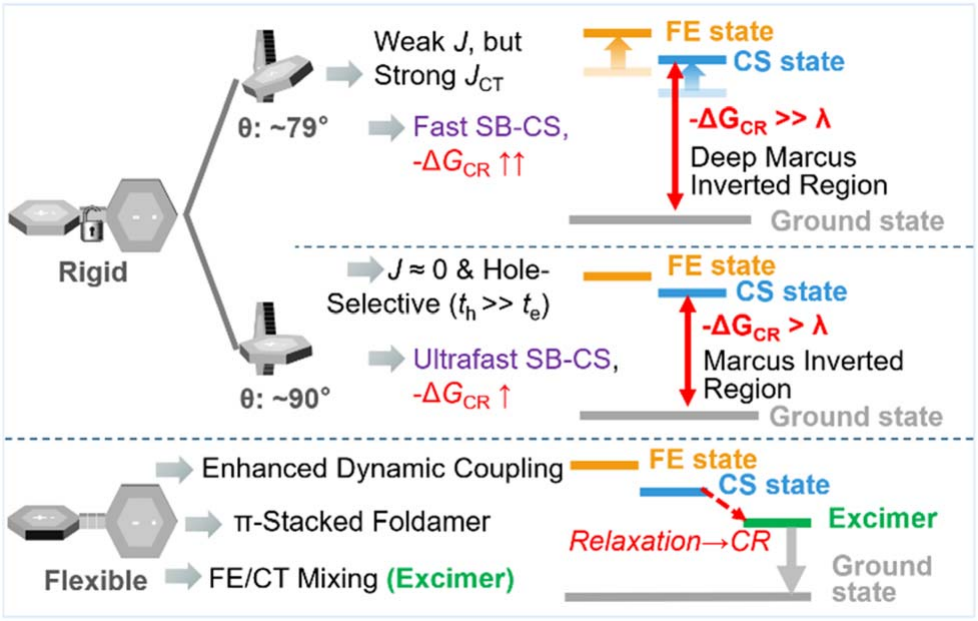
Case studies illustrating the three design axes for extending charge-separated state lifetimes.

At the molecular level, kinetic decoupling can be realized by independently tuning the key physical parameters governing electron transfer. These can be conceptualized as three largely orthogonal design axes: electronic coupling, thermodynamic driving force, and structural dynamics (Fig. 7). The interplay among these parameters ultimately determines whether a charge-separated state, once formed, can be kinetically stabilized against recombination. These three axes directly map onto a universal structure–property chain that links ground-state geometry (orthogonality and rigidity, Fig. 7) to the coupling regime, then to the excited-state pathway (SB-CS *vs.* excimer formation), and finally to the functional outcome (lifetime and efficiency).

#### 3.1.1 Electronic decoupling: controlling coupling asymmetry

Electronic coupling plays a dual role in SB-CS systems, governing both the rate of charge separation and recombination. In conventional symmetric dimers, these processes originate from similar orbital overlaps, making forward and backward electron transfer intrinsically correlated. Consequently, increasing coupling to accelerate charge separation often simultaneously accelerates recombination. In most SB-CS systems, electronic coupling falls within the 10–100 meV regime, where small changes in geometry can lead to substantial variations in transfer rates. A central design objective is therefore to break this symmetry by creating distinct electronic pathways for forward and backward transfer. This requires selectively enhancing orbital interactions that promote charge separation while suppressing those facilitating recombination.

**Fig. 8:**
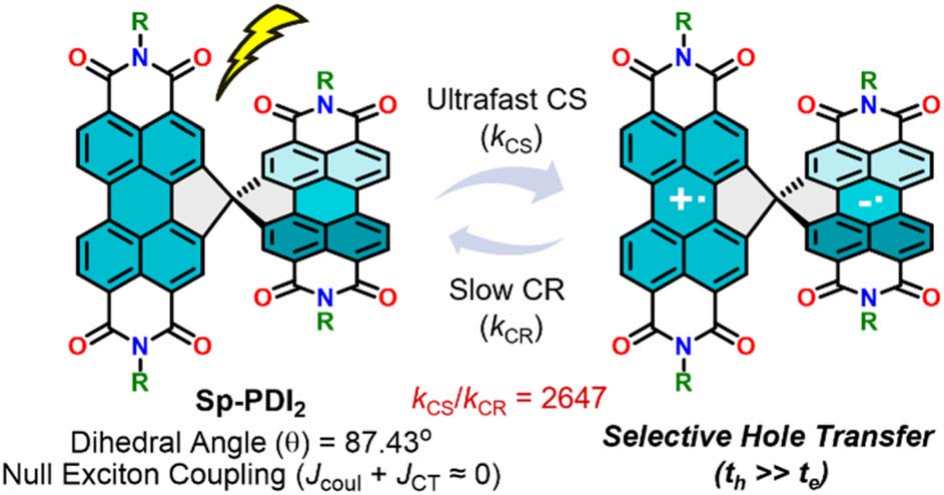
Null exciton coupling in Sp-PDI_2_ enables selective hole transfer and exceptional kinetic asymmetry.

A minimal yet compelling demonstration of this principle is provided by rigid near-orthogonal chromophore architectures (Sp-PDI_2_, Fig. 8). In such systems, ground-state excitonic coupling is effectively eliminated through geometric decoupling, while through-bond charge-transfer interactions remain operative. For example, a spiro-conjugated PDI dimer reported by Sebastian and Hariharan exhibits an exceptional *k*_CS_/*k*_CR_ ratio of 2647 in acetonitrile.^15^ This behavior originates from constructive HOMO–HOMO coupling facilitating hole transfer, combined with destructive LUMO–LUMO interference suppressing recombination, thereby intrinsically differentiating the forward and backward electronic pathways.^16^

#### 3.1.2 Thermodynamic control: navigating the energy landscape

While electronic design determines whether SB-CS can occur, thermodynamic positioning determines whether it persists. Within Marcus electron transfer theory, forward and backward transfer rates depend on the interplay between reaction free energy (Δ*G*) and reorganization energy (*λ*), defining distinct kinetic regimes.

A particularly powerful strategy is to position recombination within the Marcus inverted region, where increasing the driving force paradoxically slows the electron transfer rate. This enables thermodynamic asymmetry in which charge separation occurs with a modest driving force while recombination occurs with an excessive driving force, allowing rapid forward transfer alongside intrinsically slow back transfer. However, achieving and maintaining such inverted-regime conditions in complex assemblies remains challenging, as small environmental or structural perturbations (often on the order of ∼100 meV) can significantly alter recombination kinetics.

**Fig. 9:**
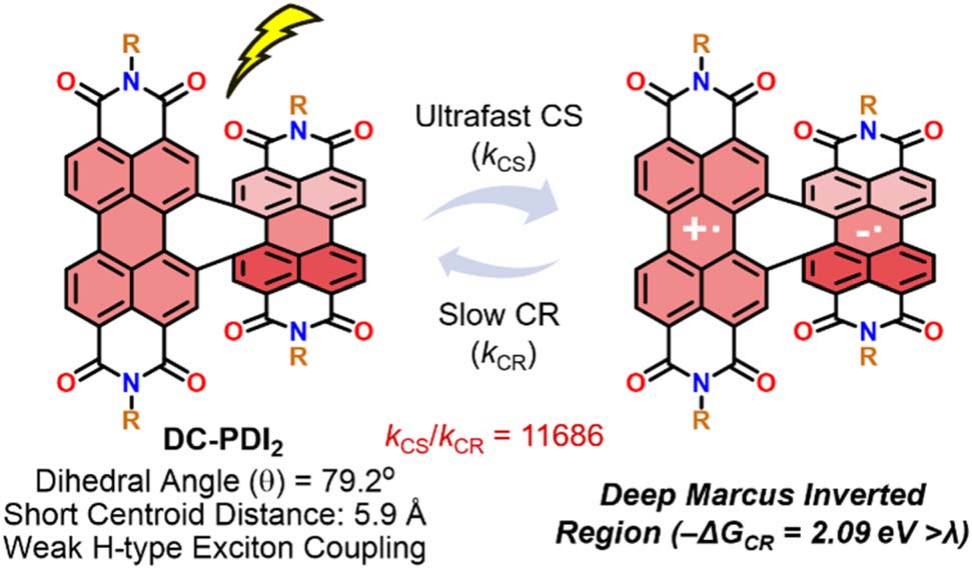
Enhanced charge-transfer coupling in DC-PDI_2_ enables ultrafast SB-CS (*τ* ≈ 0.35 ps) and slow charge recombination (*τ* ≈ 4.1 ns) due to charge recombination occurring in the deep Marcus inverted region (|Δ*G*_CR_| > *λ*), resulting in exceptional kinetic asymmetry between forward and backward processes.

Quantitative validation of this concept was provided by Sebastian and Hariharan through the design of a rigid near-orthogonal PDI dimer (DC-PDI_2_) in which recombination occurs deep within the inverted region (−Δ*G*_CR_ = 2.09 eV exceeding *λ* ≈ 0.8 eV, Fig. 9).^3*h*^ This energetic positioning produced an exceptional kinetic asymmetry, with *k*_CS_/*k*_CR_ reaching 11 686 in acetonitrile. Furthermore, modest substituent modifications altered −Δ*G*_CR_ by only ∼140 meV yet accelerated recombination by nearly an order of magnitude, experimentally confirming the strong kinetic sensitivity predicted using Marcus theory.^17^

#### 3.1.3 Structural rigidification: suppressing relaxation-driven recombination

Beyond electronic and thermodynamic considerations, molecular structural dynamics introduce a third independent control dimension. Excited-state structural relaxation frequently enables competing deactivation pathways such as excimer formation or non-radiative decay, which can rapidly quench SB-CS even when electronic coupling and energetics are favorably designed.

**Fig. 10:**
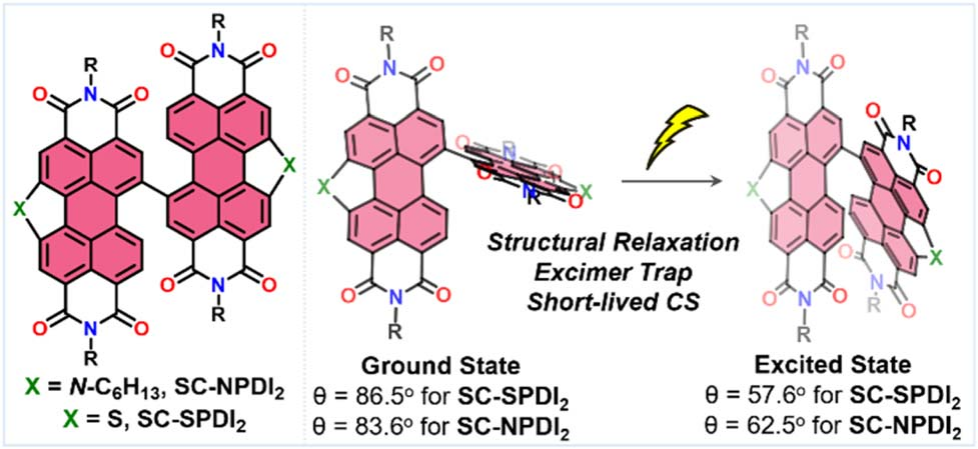
Excimer formation in flexible core-annulated PDI dimers (SC-NPDI_2_ and SC-SPDI_2_) shortens the SB-CS lifetime, highlighting rigidification as a key design principle.

Rigidification strategies suppress these pathways by locking the interchromophoric orientation and preventing conformational changes that dynamically reshape the excited-state energy landscape. A representative example comes from core-annulated PDI dimers (SC-NPDI_2_ and SC-SPDI_2_) studied by Sebastian and Hariharan, which, despite near-orthogonal ground-state geometry, possess sufficient torsional flexibility to undergo excited-state relaxation toward π-stacked foldamer conformations (Fig. 10).^18^ This structural reorganization promotes configuration mixing between Frenkel excitons and charge-transfer states, stabilizing excimer traps that act as rapid recombination funnels. In contrast, the rigid scaffolds employed in the spiro-conjugated^15^ and doubly-linked^3*h*^ architectures inherently restrict this torsional motion, preserving the near-orthogonal geometry and preventing excimer stabilization. This demonstrates that structural locking is essential for preserving the intended electronic and thermodynamic design features by avoiding relaxation-driven energetic collapse.

### 3.2 From kinetic decoupling to spatial decoupling: extending SB-CS beyond the molecular unit

Even when electronic coupling and energetics are optimized at the molecular level, charge recombination remains efficient as long as electrons and holes remain spatially proximal. This limitation highlights a fundamental constraint of treating SB-CS as a local, dimer-centered event. In functional systems, however, SB-CS should instead be viewed as a process that evolves across multiple length scales.

**Fig. 11:**
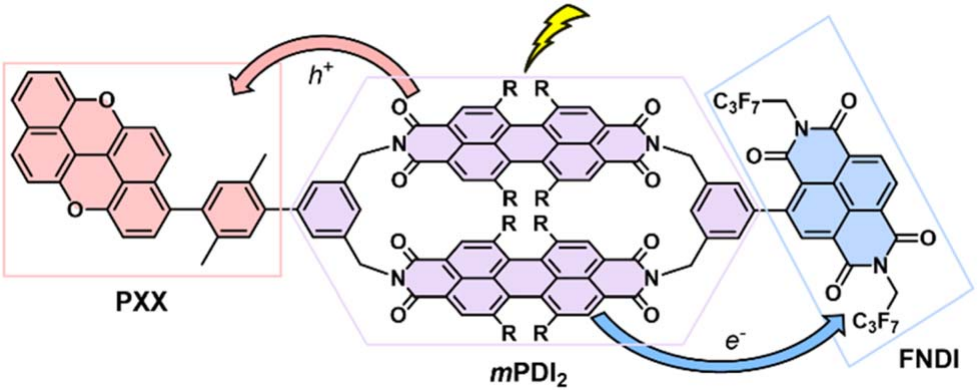
Cascade charge transfer in a PDI-based triad (mPDI_2_–PXX–FNDI) enables spatial decoupling and long-lived charge separation (*τ* ≈ 3 µs).

In this broader perspective, the dimer represents only the minimal unit for charge generation, but not the functional unit for charge utilization. Rather, functionality emerges from spatially extended networks, as exemplified by the mPDI_2_–PXX–FNDI triad reported by Bradley, Wasielewski and co-workers,^5^ in which the initially generated charge-separated state is further processed through charge transport (Fig. 11). In such architectures, multistep charge-transfer cascades, reminiscent of natural photosynthetic reaction centers, enable progressive spatial separation *via* sequential electron and/or hole transfer to secondary sites.

Importantly, this transition from local generation to nonlocal transport fundamentally changes the nature of the electronic states involved. The initially formed CT state is not itself the transport-active species; instead, interactions with the surrounding environment and molecular packing promote further charge localization, giving rise to fully charge-separated states that become distributed across a supramolecular framework and govern long-range charge migration.

Within this spatially extended regime, recombination is likewise transformed. Rather than being dictated solely by intrinsic electronic coupling within a dimer, recombination becomes a nonlocal process that depends on the spatial overlap of charges across the network. As electrons and holes migrate apart, their wavefunction overlap decreases, leading to an effective suppression of recombination and substantial extension of charge-separated state lifetimes. In such regimes, recombination rates are expected to decay exponentially with the spatial distance between the electron and hole (typically over 1–2 nm length scales). However, from a materials design perspective, this benefit is often accompanied by a structural trade-off: architectures that maximize charge separation and suppress recombination do not necessarily provide optimal pathways for efficient long-range charge transport and extraction.

However, this nonlocal strategy introduces new design constraints. Each additional transfer step may incur energetic losses, and excessive spatial separation can reduce the accessibility of charges for extraction or chemical utilization. Thus, effective system design requires balancing distance-driven stabilization with the maintenance of functional connectivity.

Overall, spatial organization introduces a distinct level of control beyond molecular energetics, governing whether charge recombination can be suppressed through nonlocal separation. Moreover, the same cascade architectures that enable spatial decoupling naturally establish energetic gradients, providing a structural basis for directional charge transport, as discussed in Section 4.1.

#### 3.2.1 Design principle: kinetic decoupling through multidimensional control

Taken together, these approaches converge on a unifying design principle: functional SB-CS systems require kinetic decoupling of charge generation from charge recombination.

This decoupling can be achieved through multidimensional control across complementary design variables: electronic coupling (differentiating orbital pathways to independently tune *k*_CS_ and *k*_CR_), thermodynamic positioning (placing forward and backward transfer in distinct kinetic regimes), structural control (preserving favorable excited-state configurations), and spatial separation (reducing recombination through controlled charge migration).

These variables are interdependent. Increasing spatial separation reduces electronic coupling, which in turn influences both forward and backward rates. Similarly, energetic tuning may alter effective coupling regimes through changes in nuclear reorganization. Successful lifetime engineering therefore requires integrated control across multiple dimensions rather than optimization along a single parameter. This multidimensional framework reframes lifetime extension not simply as slowing recombination, but as designing kinetic architectures in which charge separation and recombination are intrinsically decoupled. This framework also establishes a direct conceptual bridge to the next design challenge, namely, transforming long-lived but still localized charge-separated states into systems capable of directional charge transport.

## 4 Directionality as an emergent property: engineering vectorial charge flow from symmetric origins

While intrinsic symmetry breaking determines where charges are initially generated and lifetime engineering governs how long they persist, functional systems ultimately require control over where those charges propagate. In SB-CS systems, this represents the final design challenge: converting an initially symmetric excitation into directional (vectorial) charge transport.

Symmetry breaking does not inherently confer directionality. In an ideal symmetric dimer, charge separation produces two energetically equivalent charge-localized states, corresponding to two equally probable evolutionary pathways. Even after symmetry is locally broken, the system remains statistically isotropic, lacking any intrinsic spatial preference for charge motion. Consequently, symmetry breaking alone does not encode vectorial information, and charge migration remains unbiased in the absence of additional driving forces. This distinction is frequently overlooked, as many systems exhibit efficient symmetry breaking yet fail to generate net charge transport due to the absence of spatial bias.

Directionality does not emerge from symmetry breaking *per se*, but from spatial gradients in the energy landscape that bias charge motion along a preferred pathway. Such gradients may originate from multiple physical sources, including thermodynamic driving forces (Δ*G*), electrostatic fields (local dipoles or internal electric fields), and structural organization (molecular packing, connectivity, and dimensionality within the assembly).

The central design challenge, therefore, is not merely to induce symmetry breaking, but to encode well-defined spatial gradients that convert symmetric charge generation into biased charge flow. From this perspective, two complementary strategies can be identified: (i) thermodynamic bias introduced through energetic gradients and (ii) intrinsic electrostatic asymmetry embedded within the molecular or supramolecular architecture. These approaches represent distinct yet interconnected routes to imposing spatial asymmetry on the energy landscape, thereby transforming isotropic charge evolution into directional transport.

### 4.1 Thermodynamic bias: direction from energy gradients

The most direct route to achieving directionality is to impose an energetic gradient that biases charge migration along a preferred pathway. This is typically realized by coupling an SB-CS-active unit to secondary donor or acceptor sites with progressively aligned redox potentials, thereby establishing a downhill free-energy landscape for charge transfer.

Importantly, the cascade architectures introduced in Section 3 for lifetime extension also play a central role in this context. However, their function in directionality is fundamentally distinct from their role in lifetime engineering. Whereas lifetime extension arises primarily from spatial separation and the kinetic suppression of recombination, directionality emerges from the creation of a spatially resolved energetic gradient that defines the trajectory of charge flow.

Within such architectures, the essential function is not merely to increase charge separation distance, but to lift the degeneracy of the energy landscape and impose a directional bias. Each successive charge-transfer step is energetically favored along a specific pathway, thereby converting an initially symmetric charge generation event into vectorial charge transport. As a result, electron and hole dynamics are no longer isotropic, but instead follow predefined energetic trajectories.

This mechanism closely parallels that of natural photosynthetic reaction centers, where sequential electron-transfer steps both extend charge separation and enforce directionality through an energetically ordered manifold of states. More broadly, cascade architectures can be viewed as multifunctional design elements that simultaneously enable kinetic stabilization (*via* spatial separation) and directional control (*via* energy gradients) within a unified framework.

However, this strategy introduces an inherent trade-off. The imposition of strong energetic gradients necessarily breaks strict symmetry and shifts the system toward conventional donor–acceptor architectures. In this sense, directionality is achieved by replacing energetic degeneracy with bias, partially sacrificing the symmetry that underpins SB-CS.

Thus, thermodynamic gradients provide a robust and general route to directional charge transport. However, they do so by transitioning the system toward energetically preprogrammed pathways, rather than purely symmetry-driven processes.

### 4.2 Intrinsic electrostatic asymmetry: direction from local fields

As discussed in Section 2.3, electrostatic asymmetry can lift energetic degeneracy prior to photoexcitation, thereby enabling intrinsic symmetry breaking. The same local electrostatic fields can generate a spatially asymmetric energy landscape that biases the evolution of the charge-separated state. In assemblies such as slip-stacked chromophores with embedded dipoles or bichromophores coordinated with cations, local fields introduce electrostatic gradients that energetically differentiate nominally equivalent charge-localization pathways. Consequently, electrons and holes preferentially localize along a defined direction, even in the absence of externally imposed energetic cascades.

A key distinction of this mechanism is its temporal hierarchy. Unlike thermodynamic cascades, which enforce directionality through sequential downhill charge-transfer steps, electrostatic asymmetry operates at the earliest stages of the photophysical process. By biasing the initial charge localization event itself, directionality is encoded directly into the primary charge-separation dynamics, without requiring chemical differentiation of the constituent chromophores.

However, electrostatic asymmetry alone is generally insufficient to sustain long-range directional transport. In most systems, the magnitude of the electrostatic bias is comparable to or smaller than thermal energy at 300 K (*k*_BT_ ≈ 25 meV), limiting its ability to enforce deterministic directionality under ambient conditions. As a result, while local fields can bias charge separation, they often do not prevent recombination or enable extended charge migration, as charges remain spatially confined within chemically equivalent frameworks.

**Fig. 12:**
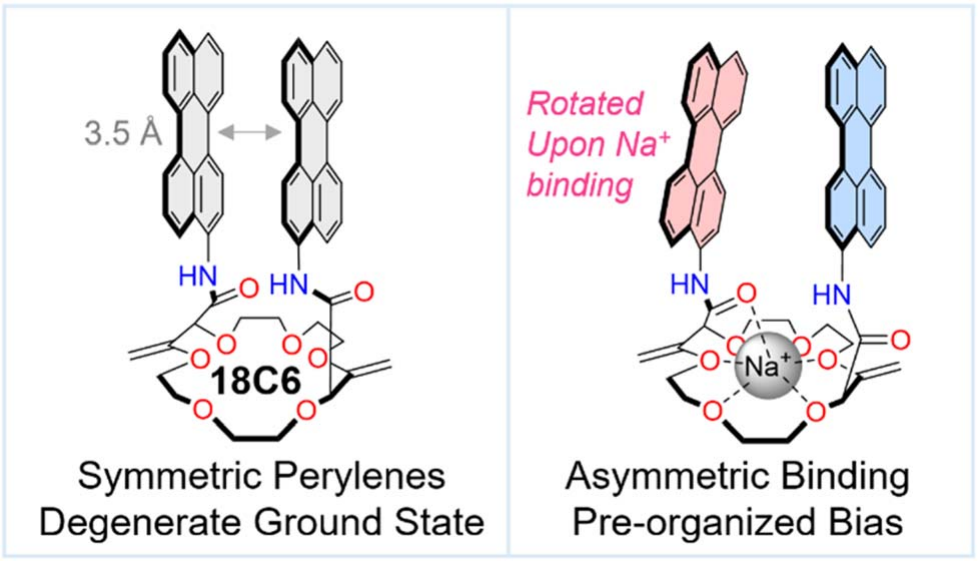
Na^+^ binding induces ground-state asymmetry in a crown-ether-linked perylene bichromophore (18C6).

This limitation underscores a critical design requirement: directional bias must be coupled to transport pathways. Electrostatic gradients can define where charges preferentially localize, but additional energetic or structural gradients are required to dictate how far and how efficiently they propagate. Experimental studies support this role of local electrostatic fields. For example, Vauthey and co-workers demonstrated that cation binding in perylene crown-ether bichromophores generates asymmetric coordination environments that lift degeneracy and bias charge separation (Fig. 12).^19^ In optimized configurations, such electrostatic asymmetry enables efficient SB-CS with extended lifetimes, highlighting how local fields can both stabilize and direct charge formation.

In this regard, these systems bear conceptual similarity to natural photosynthetic reaction centers, where local electrostatic environments guide the initial charge-separation step, while higher-order structural organization ensures subsequent directional transport.

This leads to a general design principle: electrostatic asymmetry can encode directionality without chemical differentiation, but must be integrated with energetic or structural gradients to achieve sustained, long-range vectorial charge flow.

#### 4.2.1 Design principle: from symmetry to vectorial flow

Effective directional control therefore typically arises from the cooperative interplay of multiple mechanisms. For example, intrinsic electrostatic asymmetry may define the initial direction of charge localization, thermodynamic gradients may guide subsequent migration, and extended architectures may enable spatial separation and long-range transport. In this integrated view, directionality is not a property of an isolated molecular unit, but a system-level function emerging from the coupling of molecular structure, energetics, and spatial organization.

This perspective completes the functional evolution of SB-CS systems: from charge separation (Section 2), to charge stabilization (Section 3), to directed charge utilization (Section 4). Ultimately, the central challenge is not merely to break symmetry, but to hierarchically lift and exploit it, transforming stochastic charge generation into controlled, vectorial charge flow.

## 5 Toward a hierarchical design principle for SB-CS systems

Building on this insight, we propose a unified design framework in which SB-CS is governed by three interdependent dimensions: symmetry origin, kinetic asymmetry, and spatial organization.

**Fig. 13:**
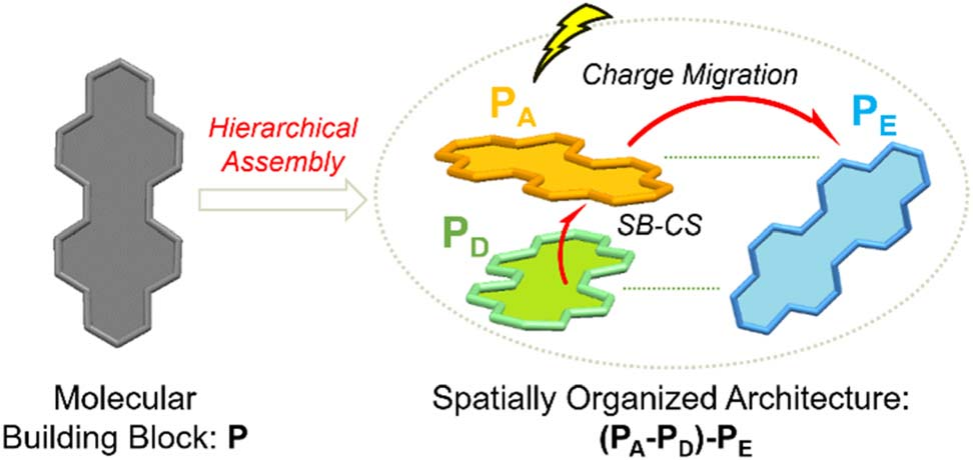
Bioinspired model: from a single molecular building block to hierarchical assemblies enabling directional, long-lived charge separation under light.

Within this perspective, one possible minimal functional motif can be represented by a (P_A_–P_D_)–P_E_ architecture (Fig. 13). The primary pair (P_A_–P_D_) consists of two chemically identical chromophores that remain strongly electronically coupled, preserving the efficient charge-generation characteristics associated with symmetry-breaking charge separation. In this conceptual framework, P_A_ and P_D_ are not intrinsically differentiated by molecular structure. Instead, asymmetry is introduced at a higher hierarchical level through interactions with a secondary element (P_E_). By interacting unequally with the two chromophores of the primary pair, P_E_ can lift degeneracy, bias charge localization, and potentially guide subsequent charge migration. In this way, strong coupling and directional bias emerge from distinct organizational levels, suggesting a possible route toward combining the efficient charge-generation characteristics of SB-CS systems with the directional control traditionally associated with donor–acceptor architectures. This conceptual framework can be mapped onto three practical design parameters that serve as actionable “design knobs” for molecular and supramolecular engineering:

(i) Electronic coupling (*J*): strong intra-unit coupling (typically exceeding thermal energy at 300 K, *k*_BT_ ≈ 25 meV) is required to enable efficient symmetry-breaking charge generation. In contrast, weaker and asymmetric inter-unit coupling differentiates forward and backward charge-transfer pathways, facilitating kinetic decoupling.

(ii) Free-energy landscape (Δ*G*): charge separation is ideally positioned near thermoneutral or slightly downhill conditions, whereas recombination should be driven into the inverted or weak-coupling regime to suppress back electron transfer. Optimal operating windows are often within ±0.1–0.2 eV of thermoneutrality.

(iii) Spatial gradient (distance and geometry): molecular arrangement controls both the decay of electronic coupling and the emergence of directional bias, thereby linking structural organization to transport functionality.

Notably, this minimal three-body motif represents the smallest architectural unit in which symmetry breaking, kinetic decoupling, and directional bias can be simultaneously encoded. However, these parameters are intrinsically interdependent and must be co-optimized within a hierarchical architecture. For example, increasing electronic coupling to promote efficient charge separation must be balanced by energetic or spatial differentiation to prevent rapid recombination, while introducing gradients to enforce directionality must preserve accessibility for charge extraction and utilization.

**Fig. 14:**
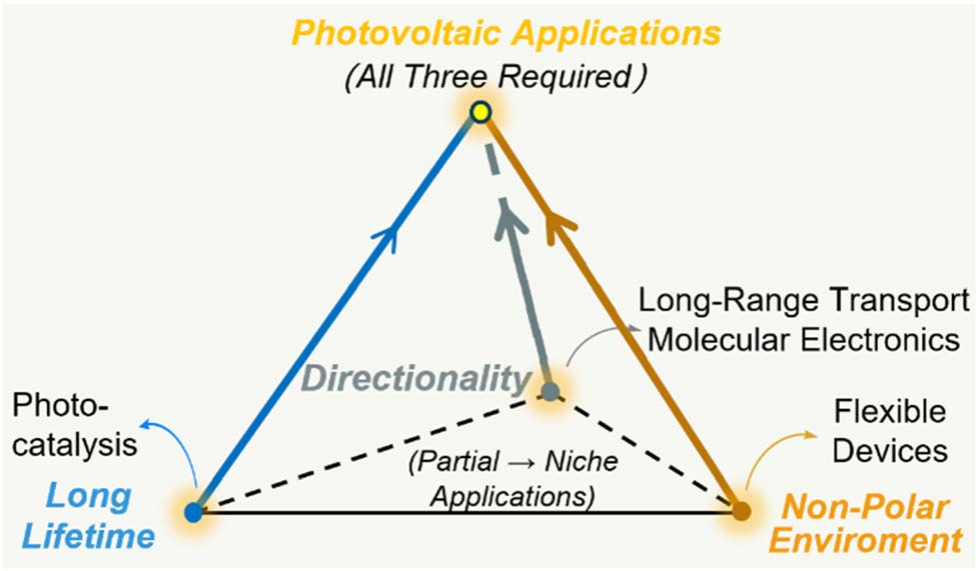
A tetrahedral framework illustrating the stepwise integration of three fundamental SB-CS design targets toward high-efficiency photovoltaics.

From this perspective, functional SB-CS systems do not emerge from optimizing a single parameter, but from aligning multiple design variables within an integrated architectural framework. Importantly, this framework assumes that electronic coupling, energetics, and spatial organization can be tuned with a degree of independence. In practice, however, these variables are often strongly coupled, making their simultaneous optimization a central challenge in SB-CS design. The resulting design space is therefore best viewed as a multidimensional landscape in which charge generation, stabilization, and transport must be balanced within a hierarchical architecture (Fig. 14).

## 6 Outlook: from design principles to functional landscapes

Moving from molecular design principles to device-relevant functional materials introduces additional layers of complexity that current frameworks have yet to fully address.

Meeting this challenge will require a tightly integrated effort across theory, synthesis, and time-resolved characterization. On the theoretical side, future progress depends on multiscale models capable of bridging electronic structure, nuclear motion, mesoscale organization, and device-level operation. Such approaches must move beyond static energy-level descriptions to explicitly incorporate dynamic disorder, vibronic coupling, non-equilibrium relaxation, and interfacial charge transfer. Only through this expanded framework can one understand how local molecular interactions propagate into macroscopic charge separation and transport behavior. In parallel, ultrafast spectroscopy spanning femtosecond to microsecond timescales will be indispensable for resolving the coupled electronic and structural dynamics that govern SB-CS across multiple temporal regimes. Complementary advances in molecular and supramolecular synthesis will be equally essential, enabling architectures with deliberately encoded coupling motifs, controlled asymmetry, and programmable packing landscapes.

More broadly, SB-CS should be viewed not as a single mechanistic solution, but as a modular design paradigm spanning multiple functional landscapes (Fig. 14). Different applications occupy distinct regions of SB-CS design space, each demanding a different balance of constraints. In photovoltaics, efficient operation requires the simultaneous optimization of charge generation, long-range transport, and interfacial extraction—objectives that are often mutually antagonistic. In photocatalysis, by contrast, the central requirement may be the controlled persistence of long-lived charge-separated states rather than rapid transport. Emerging opportunities in spin chemistry^20^ and quantum information science exploit the coherent radical-pair states generated by symmetry breaking, where spin selectivity, coherence lifetimes, and controllable spin evolution become key design targets. From this perspective, the widely discussed limitations of SB-CS in solar-energy conversion should not be interpreted as an intrinsic weakness of the mechanism itself, but rather as evidence that different applications demand fundamentally different optimization criteria.

A decisive step forward will be extending SB-CS from well-defined molecular assemblies to device-relevant functional materials. This transition introduces additional layers of complexity, including heterogeneous interfaces, mesoscale disorder, morphological variability, and long-range packing heterogeneity. Such features can suppress, obscure, or in some cases even enhance SB-CS behavior. Future success will therefore depend on unifying molecular precision with materials-level control, encompassing not only chemical structure, but also morphology, dimensionality, defect landscapes, and interfacial organization.

Within this broader context, SB-CS may be redefined as a multiscale design problem in which symmetry is not merely broken, but intentionally encoded, amplified, and propagated across hierarchical architectures. The progression from symmetry → bias → flow, developed in this Perspective, provides a conceptual scaffold for this transformation by linking molecular-scale interactions to emergent system-level function. Directionality, stability, and useful work arise not from any single parameter, but from the coordinated alignment of multiple structural, energetic, and dynamical variables within an integrated landscape.

Looking ahead, one of the most transformative goals will be the construction of predictive SB-CS design maps that connect molecular structure, electronic coupling, supramolecular organization, and environmental context to measurable functionality. Such maps would enable the rational navigation of SB-CS design space, guiding the creation of systems in which charges are generated efficiently, stabilized selectively, directed purposefully, and ultimately harvested productively.

In this sense, the future of SB-CS does not lie in refining isolated molecular mechanisms, but in integrating them into coherent, hierarchical strategies that span length scales from molecules to materials to devices. By embracing this multiscale vision, SB-CS can evolve from a model photophysical curiosity into a general framework for engineering functional charge flow in complex molecular systems, with implications extending from solar-energy conversion to catalysis, spin technologies, and molecular information science.

## Author contributions

H.-J. Z. and J. L. wrote the initial draft. L. W. and J. W. participated in literature surveys and discussions, designed and prepared the figures, and contributed to manuscript editing. J. L. and D. K. conceived the conceptual framework, and revised the manuscript with H.-J. Z. All authors approved the final version.

## Conflicts of interest

There are no conflicts to declare.

## Data Availability

This Perspective article does not report new experimental or computational data. All data discussed are taken from previously published literature and are appropriately cited in the text. The figures and schematics presented in this article are original conceptual illustrations included within the main text. No datasets or code were generated in this work.
